
JOURNAL INFORMATION
==============================
NLM Title Abbreviation: ScientificWorldJournal
Journal ID: ScientificWorldJournal
NLM Journal ID: 101131163
Journal ID: TSWJ

The Scientific World Journal

ISSN: 2356-6140
EISSN: 1537-744X
Publisher: Wiley

ARTICLE INFORMATION
==============================
PMCID: PMC5956429
PMID: 15258671
DOI: 10.1100/tsw.2004.102
Article ID: 198475
Article version: 1
Subjects: Case Study

Primary Infertility and Azoospermia Due to Congenital Bilateral Absent Vas Deferens in the Presence of a Solitary Kidney

Karim Amer
Institute of Urology, London
Academic Editor: Anthony Atala

Publication date: 2004
Electronic publication date: 2004 Jun 18
Volume: 4
First page: 458
Last page: 459
Copyright: Copyright © 2004 Amer Karim.
Copyright year: 2004
License: This is an open access article distributed under the Creative Commons Attribution License, which permits unrestricted use, distribution, and reproduction in any medium, provided the original work is properly cited.
License URL: https://creativecommons.org/licenses/by/3.0/

==============================
Case Study
*TheScientificWorldJOURNAL* (2004) 4, 458–459
ISSN 1537-744X; DOI 10.1100/tsw.2004.102

### Primary Infertility and Azoospermia Due to Congenital Bilateral Absent Vas Deferens in the Presence of a Solitary Kidney

Amer Karim
*Senior House Surgeon, Institute of Urology, London*

**E-mail:** amerkarim@e320.freeserve.co.uk

*Published June 18, 2004*

**DOMAIN:** urology

### CASE REPORT

A 27-year-old gentleman referred with azoospermia and a normal hormone profile was shown on MRI examination to have an absent left ureter and seminal vesicle (Fig. 1). His subsequent IVU showed that his left kidney was also absent (Fig. 2).

He was diagnosed with congenital absence of vas deferens (CAVD) and was successfully treated with percutaneous sperm aspiration and subsequent IVF therapy.

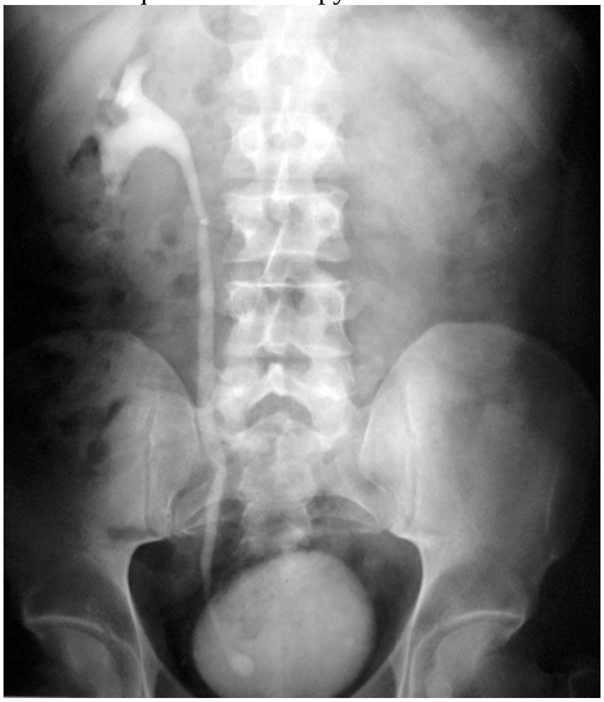

**FIGURE 1**. IVU demonstrating single right kidney.

Author address: 17 Rampart Walk, Dorchester, Dorset, DT1 2QZ;
Tel: +44 1305266415/Fax: +44 1305266415
©2004 with author.

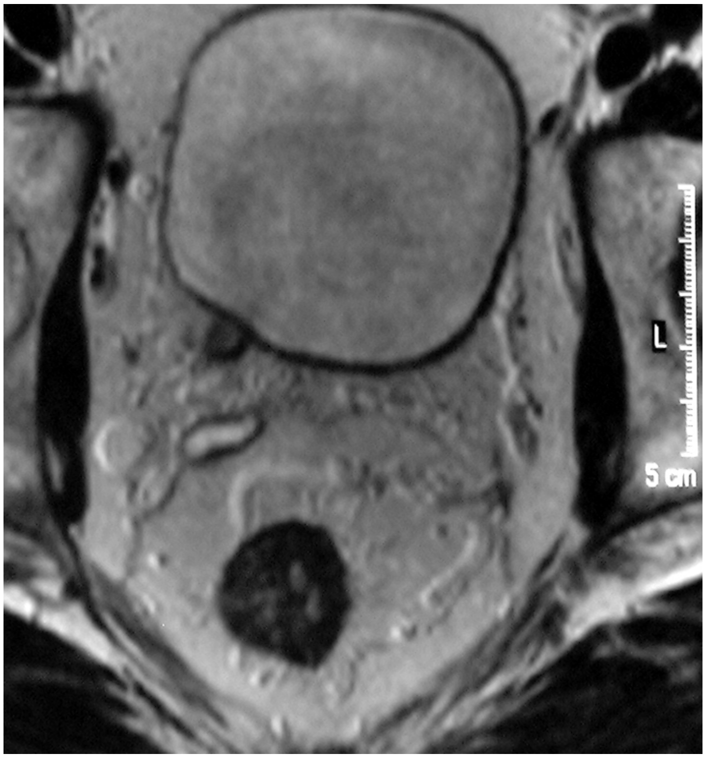

**FIGURE 2**. MRI demonstrating absent left seminal vesicle and ureter.

### COMMENTS

CAVD is a syndrome in which a portion or all of the vas deferens, epididymis, and seminal vesicles are absent, either unilaterally or bilaterally[1]. It is the cause for approximately 1–2% of male infertility and 10% of ductal obstruction[2]. CAVD is an inherited condition, associated with heterozygous presence of one of the gene anomalies causing cystic fibrosis on chromosome. As in this case, 15% of patients with CAVD will also have a missing or ectopic kidney. Testicular size is usually normal, the distended caput epididymis may be palpable (Bayle's sign), and no vas is palpable within the spermatic cord. Spermatogenesis is usually normal.

### REFERENCES

1. Shafik, A. (1996) Electrovasogram in patients with obstructive azoospermia and absent vas deferens. *World J. Urol*. **14(6),** 393–396.
2. Della Morte, E., Capellano, F., and Cantanzo, F. (1996) Role and current significance of EDVG (epididymography plus vasography) in obstructive azoospermia. *Arch. Ital. Urol. Androl*. **68(5),** 367–372. [Italian]

**This article should be referenced as follows:**

Karim, A. (2004) Primary infertility and azoospermia due to congenital bilateral absent vas deferens in the presence of a solitary kidney. *TheScientificWorldJOURNAL* **4**, 458–459.

**Handling Editor:**

Anthony Atala, Principle Editor for *Urology* — a domain of *TheScientificWorldJOURNAL*.
